# Relationship between Cataract Surgery and Mortality in Elderly Patients with Cataract: Nationwide Population-Based Cohort Study in South Korea

**DOI:** 10.3390/jpm11111128

**Published:** 2021-11-01

**Authors:** Jae-Yong Kim, Ho-Seok Chung, Ji-Sung Lee, Hun Lee, Hungwon Tchah

**Affiliations:** 1 Asan Medical Center, Department of Ophthalmology, University of Ulsan College of Medicine, Seoul 05505, Korea; jykim2311@amc.seoul.kr; 2 Department of Ophthalmology, Dankook University Hospital, Dankook University College of Medicine, Cheonan 31116, Korea; chunghoseok@gmail.com; 3 Clinical Research Center, Asan Medical Center, Asan Institute for Life Sciences, University of Ulsan College of Medicine, Seoul 05505, Korea; totoro96a@gmail.com; 4 Asan Medical Center, Department of Clinical Epidemiology and Biostatistics, University of Ulsan College of Medicine, Seoul 05505, Korea

**Keywords:** cataract, cataract surgery, all-cause mortality, cause-specific mortality, NHIS-senior database

## Abstract

We aimed to investigate the relationship between cataract surgery and all-cause and cause-specific mortality in Korean elderly patients with cataract using the Korean National Health Insurance Service-Senior cohort database. Elderly patients (≥60 years) diagnosed with cataract from 2002 through 2012 were included. The baseline characteristics included demographics and systemic and ocular comorbidities. Adjusted Cox regression models with time-varying covariates for cataract surgery were used to assess the relationship between cataract surgery and mortality. The study cohort included 241,062 patients, of whom 127,941 were in the cataract surgery group and 113,121 were in the cataract diagnosis group. The incidence of all-cause mortality was 3.62 deaths/100 person-years and 3.19 deaths/100 person-years in the cataract surgery and cataract diagnosis groups, respectively. Cataract surgery was associated with a decreased hazard of all-cause mortality after adjusting for demographics as well as systemic and ocular comorbidities (hazard ratio (HR), 0.93; *p* < 0.001). A protective association was noted between cataract surgery and mortality from vascular (HR, 0.92; *p* < 0.001) or neurologic (HR, 0.64; *p* < 0.001) causes. Patients with cataract who were 85 years of age and older, women, those who had lower income, and a Charlson comorbidity index score of 5 or more, or those without glaucoma revealed the largest reductions in mortality hazards resulting from cataract surgery.

## 1. Introduction

Cataract surgery has been known as the well-established treatment for visually significant cataract, which is the leading cause of visual loss among adults globally [1,2]. Nonetheless, it is uncertain whether vision improvement after cataract surgery could subsequently improve survival outcomes in the Korean elderly population [3,4,5,6].

Two previously published cohort studies demonstrated that cataract surgery was associated with a decreased mortality in cataract patients [7,8]. Another study reported that cataract surgery was associated with a decreased all-cause mortality in cataract patients from the US Medicare population [9]. Those studies were overall based on the assumption that association can be mediated by functional independence and health status improvement after surgery. On the other hand, there are recently published studies showing conflicting findings. In one study presenting the relationship of cataract surgery with total and cause-specific mortality, cataract surgery was associated with increased total and cause-specific mortality with the exception of neurologic causes in older women with cataract from the Women’s Health Initiative [10]. However, it is uncertain whether those reported findings are generalizable to the Korean elderly population.

Given the contradictory findings in previous studies from other countries, it would be necessary to investigate the relationship of cataract surgery with total and cause-specific mortality in the Korean elderly population. Furthermore, there have been no previous studies investigating the relationship between cataract surgery and mortality in Korean elderly patients with cataract. Therefore, we aimed to investigate the relationship between cataract surgery and total and cause-specific mortality in the Korean elderly population using data from a nationwide cohort, the Korean National Health Insurance Service-Senior cohort (NHIS-Senior) database, which represents the entire elderly Korean population.

## 2. Materials and Methods

### 2.1. Study Design and Source of Data

This population-based retrospective cohort study used data from the Korean NHIS-Senior database from 2002 to 2015. The database comprises the data of 558,147 individuals randomly sampled from 10% of the approximately 5.5 million Korean individuals aged >60 years and is compiled by the Korean NHIS [11]. All participants included in the NHIS-Senior database were followed up until 2015 unless they were disqualified for health coverage reasons, such as death or emigration. The NHIS-Senior database comprises patient data, such as age, gender, and hospital and pharmacy visit information, including disease diagnoses, status, procedures, and prescribed medications. Patient healthcare records are not duplicated because all Korean residents receive a unique identification number at birth. The KNHIS uses the KCD codes, which is a system similar to the International Classification of Diseases, and the KEDI codes [6]. This cohort can be used for national healthcare evidence-based analyses for statistically representing elderly Koreans [12,13]. As the NHIS-Senior database comprises publicly opened data, the Institutional Review Board of the Asan Medical Center and the University of Ulsan College of Medicine approved the waiver of reviewing this study (AMC 2019-1630).

### 2.2. Study Population

We selected the target population among those who were included in the NHIS-Senior database between 1 January 2002 and 31 December 2015 (*n* = 558,147). The inclusion criterion was the presence of at least one NHIS record between 1 January 2002 and 31 December 2012 (*n* = 284,472) with the following conditions: a KCD-7 code for cataract and age 60 years or older during this period (Table S1). The eligible subjects were classified into a cataract surgery group and a cataract diagnosis group (non-surgery group). Patients with the following characteristics were excluded: age younger than 60 years, codes for infantile and juvenile cataract, traumatic cataract, cataract secondary to intraocular surgery, after cataract, other disorders of lens, aphakia, dislocation of lens, and other specified disorders of lens.

### 2.3. Exposure

The exposure of interest was cataract surgery. Initially, we applied the wash-out period of between 1 January and 31 December 2002 to reduce the potential impact of surveillance bias. The cataract surgery group consisted of all participants with an ICD-10 diagnosis code for cataract and a KEDI code for cataract surgery. For each patient, cataract surgery was defined as the simultaneous claim of either “extracapsular or intracapsular extraction” (KEDI code S5111) or “phacoemulsification” (KEDI code S5119) and “primary intraocular lens implantation” (KEDI code S5117) on the same day (KEDI codes S5111 + S5117, S5119 + S5117, S5113 + S5117) (Table S1). Procedures in combination with vitrectomy or glaucoma surgery were excluded. The cataract diagnosis group was the unexposed group and consisted of all participants with an ICD-10 diagnosis code for cataract but without a KEDI code for cataract surgery. Patients were followed up starting from the date of the earliest cataract diagnosis.

### 2.4. Outcome

The primary outcome of interest was total and cause-specific mortality at any time from patient inclusion in the study until the end of the study on 31 December 2015. Mortality status was ascertained from an indicator variable in the NHIS-Senior database, which contains information on total and cause-specific mortality. In this study, causes of death were grouped into cancer, vascular, pulmonary, neurologic, infectious, accident, or trauma-related conditions (Table S2).

For the cataract diagnosis group, time to death was calculated as the number of days from cataract diagnosis to death [10]. For the cataract surgery group, the time from the diagnosis of cataract to surgery was included in the follow-up time of the cataract diagnosis group, and the time post cataract surgery was included in the follow-up time of the cataract surgery group [10]. Participants without a recorded death were censored on the last known date or on 31 December 2015 if they were still enrolled.

### 2.5. Covariates

Demographics included age at the time of cataract diagnosis, gender, residence, and income level; the residential area was divided into metropolitan (Seoul and large city) and provincial regions (small city and rural area) according to the administrative unit of Korea. Household income was categorized as either below or above the 20th percentile of national income.

Both systemic and ocular comorbidities were included as covariates and were assessed at the time of diagnosis for the cataract surgery and cataract diagnosis groups (Table S3). The CCI score, which is a weighted index of systemic disease burden based on the presence or absence of 17 systemic comorbidities, was used as a covariate [14]. The higher the CCI score, the higher the burden of systemic disease [14]. Patients were assigned a CCI score of between 0 and 6 that can be used to predict the risk of 1-year mortality. Diseases in the CCI included myocardial infarction, congestive heart failure, peripheral vascular disease, cerebrovascular disease, dementia, chronic pulmonary disease, rheumatologic disease, peptic ulcer disease, cirrhosis, hepatic failure, immunosuppression, diabetes mellitus (DM) with or without complications, hemiplegia or paraplegia, chronic renal disease, malignant neoplasms, multiple myeloma or leukemia, lymphomas, metastatic solid tumor, and AIDS [9].

Ocular comorbidities included glaucoma (KCD H40, H42), age-related macular degeneration (KCD H35.30, H35.31, H35.39), DM with ophthalmic manifestations (KCD E10.3, E11.3, E12.3, E13.3, E14.3), and severe cataract (KCD H25.2, H25.1). The presence of severe cataract was recognized as an indicator for poor vision because visual acuity data were not available [10]. Patients with diagnosis codes for Morgagnian-type senile cataract and senile nuclear cataract were assigned to have severe cataract [9,10].

### 2.6. Statistical Analysis

Cox regression models with time-varying covariate for cataract surgery were used to evaluate covariate-adjusted relationship from cataract surgery and time to death due to any cause and from cataract surgery and time to death attributed to cancer, vascular, pulmonary, neurologic, infectious, accident, or trauma-related conditions. We used two models of adjustment to account for potential confounding factors. Model 1 was adjusted for age (<70, 70–74, 75–79, 80–84, and ≥85 years) and gender. Model 2 was further adjusted for CCI and ocular comorbidities. Moreover, a subgroup analysis was performed to investigate the effects of age, gender, residence, income level, CCI score, glaucoma, age-related macular degeneration, DM with ophthalmic manifestations, and severe cataract on cataract surgery-related overall mortality.

All statistical analyses were conducted using the SAS software version 9.4 (SAS Institute, Cary, NC, United States). Statistical significance was considered at two-sided *p* < 0.05. ASD was used to compare the baseline characteristics. An ASD of >0.1 is considered clinically meaningful [15].

## 3. Results

### 3.1. Baseline Characteristics

The baseline characteristics are summarized in Table 1. The study cohort included 241,062 patients (mean (SD) age, 72.6 (6.1) years), of whom 127,491 were in the cataract surgery group and 113,121 were in the cataract diagnosis group. The largest proportion of patients in both groups were aged less than 70 years at the time of cataract diagnosis. The older population over 80 years of age had a tendency not to undergo cataract surgery (absolute standardized differences, ASD = 0.1107). There were significantly more females than males in the cataract surgery group (ASD = 0.1175). In terms of existence of ocular comorbidity, a higher proportion of ocular comorbidity was found in the cataract surgery group (53.6% in the cataract surgery and 41.3% in the cataract diagnosis groups, ASD = 0.2482). Specifically, the incidence of severe cataract or glaucoma was significantly higher in the cataract surgery group (ASD = 0.2381 for severe cataract and ASD = 0.1895 for glaucoma, respectively). Glaucoma was the most common ocular comorbidity in both groups (35.5% in the cataract surgery and 26.8% in the cataract diagnosis groups).

### 3.2. Mortality Incidence

Table 2 shows the results for mortality rates in the Korean elderly patients with cataract by surgery status. The crude incidence of mortality was 3.62 deaths/100 person-years in the cataract surgery group and 3.19 deaths/100 person-years in the cataract diagnosis group. There was a significant difference in hazard ratio (HR) of all-cause and cause-specific mortality (cancer, pulmonary, neurologic, infectious, accident, or trauma) between the cataract surgery and cataract diagnosis groups in the unadjusted model (*p* < 0.001 for all-cause, pulmonary, neurologic, infectious, accident or trauma, and *p* = 0.010 for cancer) (Table 3). Cataract surgery was associated with an increased all-cause mortality in the unadjusted model (HR, 1.03; 95% confidence interval (CI), 1.01–1.05, *p* < 0.001). However, cataract surgery was associated with a decreased all-cause mortality after adjusting for age and gender (HR, 0.95; 95% CI, 0.94-0.97, *p* < 0.001). Furthermore, this protective association was stronger after adjusting for demographics, Charlson comorbidity index (CCI), and ocular comorbidities (HR, 0.93; 95% CI, 0.92–0.95, *p* < 0.001).

There was a relationship between cataract surgery and decreased hazard for vascular causes of death after adjusting for age and gender (HR, 0.93; 95% CI, 0.90–0.96, *p* < 0.001), whereas there was no relationship between cataract surgery and vascular causes of death in the unadjusted model (HR, 1.02; 95% CI, 0.98–1.05, *p* = 0.337). This protective association between cataract surgery and mortality from vascular causes was slightly stronger after adjusting for demographics, CCI, and ocular comorbidities (HR, 0.92; 95% CI, 0.89–0.95, *p* < 0.001).

In addition, there was a relationship between cataract surgery and decreased hazard for neurologic causes of death in the unadjusted model (HR, 0.71; 95% CI, 0.65–0.78, *p* < 0.001). After adjusting for age and gender, the protective association between cataract surgery and mortality from neurologic causes was increased (HR, 0.66; 95% CI, 0.60–0.72, *p* < 0.001). After adjusting for demographics, CCI, and ocular comorbidities, a stronger protective association was observed (HR, 0.64; 95% CI, 0.58–0.71, *p* < 0.001).

Interestingly, cataract surgery was associated with increased hazards for infectious or accidental (traumatic) causes of death in the fully adjusted model accounting for demographics, CCI, and ocular comorbidities (HR, 1.12; 95% CI, 1.01–1.24, *p* = 0.034 for infectious causes of death, and HR, 1.10; 95% CI, 1.03–1.17, *p* = 0.006 for accidental or traumatic causes of death) and in the unadjusted model. On the other hand, there was no relationship between cataract surgery and mortality from cancer and pulmonary causes after adjustment.

According to the subgroup analysis evaluating factors that could affect the cataract surgery-related mortality, there were significant interactions for the age × cataract surgery, gender × cataract surgery, income × cataract surgery, CCI score × cataract surgery, and glaucoma × cataract surgery interaction terms in separate fully adjusted models (*p* < 0.001 for age, gender, CCI score, and the presence or absence of glaucoma, and *p* = 0.006 for income) (Table 4). Regarding age, there was a protective association between cataract surgery and all-cause mortality for patients of 75 years of age and older. The strongest protective association was observed in patients of 85 years of age and older, with a 25% lower hazard of mortality for patients with cataract surgery than that for those without cataract surgery (HR, 0.75; 95% CI, 0.71–0.79, *p* < 0.001). Women demonstrated a stronger protective association between cataract surgery and all-cause mortality than men, with a 12% lower hazard of mortality in women with cataract surgery than that in women without cataract surgery (HR, 0.88; 95% CI, 0.86–0.90, *p* < 0.001). Concerning the income level, patients with a lower income showed a stronger protective association between cataract surgery and all-cause mortality than those with a higher income, with a 10% lower hazard of mortality for lower-income patients with cataract surgery than that for lower-income patients without cataract surgery (HR, 0.90; 95% CI, 0.87–0.93, *p* < 0.001). Both patients with lower and higher incomes demonstrated the protective association between cataract surgery and all-cause mortality. Patients with a CCI score of 4 or more demonstrated the protective relationship between cataract surgery and all-cause mortality. Moreover, patients with a CCI score of 5 or more demonstrated the strongest protective association, with a 20% lower hazard of mortality in patients with a CCI score of 5 or more who underwent cataract surgery than that in those with a CCI score of 5 or more who did not undergo cataract surgery (HR, 0.80; 95% CI, 0.78–0.83, *p* < 0.001). For ocular comorbidity, non-glaucoma patients who underwent cataract surgery had a 9% lower hazard of all-cause mortality than non-glaucoma patients who did not undergo surgery (HR, 0.91; 95% CI, 0.89–0.93, *p* < 0.001). A lower likelihood of cataract surgery-related death was associated with age over 75 years at the time of cataract diagnosis, female gender, lower income, having a CCI score of 4 or more, and having no glaucoma (Table 4).

## 4. Discussion

Cataract surgery is the most frequently performed surgical procedure in South Korea according to the KNHIS [6]. Although there have been several studies that evaluate the relationship between cataract surgery and mortality from other countries, it is uncertain whether findings from other countries are generalizable to the Korean population. Moreover, there has been no published study evaluating the relationship between cataract surgery and mortality in the Korean elderly population using the NHIS-Senior database. Given these strengths of the NHIS database and the need for further understanding of the relationship of cataract surgery with total and cause-specific mortality, we aim to investigate the relationship between cataract surgery and total and cause-specific mortality in the Korean elderly population.

In this nationwide cohort study, Korean elderly population with cataract who underwent cataract surgery showed a decreased hazard of all-cause mortality when compared with those who did not undergo surgery after adjusting for demographics as well as systemic and ocular comorbidities. Cataract surgery was associated with a borderline increased hazard of mortality in the unadjusted model (HR, 1.03; 95% CI, 1.01–1.05) but a decreased hazard of mortality in the adjusted model accounting for age and sex (HR, 0.95; 95% CI, 0.94–0.97). This protective association increased after additionally adjusting for systemic and ocular comorbidities; patients with cataract surgery had a 7% reduced adjusted hazard of mortality when compared with those without surgery. Moreover, the elderly population after cataract surgery had a lower risk of death due to vascular and neurologic conditions than those with cataract who did not undergo cataract surgery. The protective association between cataract surgery and mortality seemed to be affected by age, gender, income, CCI score, and the presence or absence of glaucoma. Patients with cataract who were 85 years of age and older, women, those who had lower income and a severe burden of systemic disease as measured by the CCI, or those without glaucoma revealed the largest reductions in mortality hazards resulting from cataract surgery. Interestingly, the proportion of population over 80 years of age was lower, and the proportion of females was higher in the cataract surgery group than in the cataract diagnosis group. Additionally, the cataract surgery group contained higher proportions of populations with ocular comorbidities, severe cataract, and glaucoma, suggesting that cataract surgery was performed more frequently and earlier because of frequent visits for follow-up of ocular comorbidities other than cataracts.

One previous study from the United States population reported that cataract surgery was associated with a decreased all-cause mortality in cataract patients after adjusting for demographics and systemic and ocular comorbidities [9]. Two previously published reports conducted in Western Sydney, Australia also demonstrated that cataract surgery was associated with decreased all-cause mortality in patients with cataract [7,8]. In accordance with these results, our results showed that cataract surgery was associated with a decreased all-cause and cause-specific mortality, especially vascular and neurologic causes, after adjusting for demographics, and systemic and ocular comorbidities in the Korean elderly population.

We hypothesized that cataract surgery can be protective against all-cause mortality via the improvement in overall function from decreased fracture and accidents, improved mental health, and increased social and physical activities [9]. Both improvements in quality of life and reduction in depressive symptoms after surgery can also make a contribution to the protective association [16,17].

Several studies demonstrated that patients report higher scores on cognition assessments after cataract surgery [16,18,19,20]. In one recent study that evaluated the effects of cognitive performance and visual acuity on mortality, impairment in cognitive performance, and vision increased the odds for mortality [21]. Among the cognitive impaired elderly population, impairment in vision predicted nearly a three-fold increased risk of all-cause mortality (HR, 2.74; 95% CI, 2.02–3.70) and nearly a four-fold higher risk of non-cardiovascular disease/non-cancer mortality (HR, 3.72; 95% CI, 2.30–6.00) compared to having neither impairment [21]. Our results, showing a protective association between cataract surgery and mortality from neurologic causes, were in line with those studies. This protective relationship increased after fully adjusting for demographics and systemic and ocular comorbidities, and patients with cataract surgery had almost a 30% reduced adjusted hazard of mortality compared with those without cataract surgery. Although we did not investigate the relationship between neurologic diseases and eventual mortality before and after cataract surgery, we suggest that an improvement in cognitive performance and vision after cataract surgery plays a crucial role in the decreased mortality from neurologic causes.

In the present study, we demonstrated a protective association between cataract surgery and mortality from vascular causes after adjusting for demographics and systemic and ocular comorbidities. Cardiovascular risk factors that were associated with cataract and cataract surgery include hypertension, diabetes, hypercholesterolemia, and high body mass index (BMI) [22,23,24]. Meta-analyses of cohort studies showed that hypertension is related to incident cataract, especially posterior subcapsular cataract [22,23]. Cataract surgery is not only more prevalent but also performed at younger age in a high cardiovascular risk cohort [25]. Patients being less than 65 years old and taking hypertension medication showed a higher incidence of cataract surgery, and angina history was associated with a higher incidence of cataract surgery [26,27]. Although there have been no studies evaluating the relationship between cataract surgery and mortality from vascular causes, we suggest that cataract surgery can be protective against mortality from vascular causes, possibly via an increased ability to receive routine medical care, to take medications properly, and to maintain physical activities secondary to the vision improvement after cataract surgery. Further study on the inter-relationship among cataract surgery, cardiovascular disease, and disease-related mortality is needed to investigate the mechanisms underlying the relationship between cataract surgery and mortality from vascular causes.

In a recent published study that evaluated the relationship between cataract surgery and all-cause and cause-specific mortality in older women with cataract, cataract surgery was associated with increased all-cause and cause-specific mortality (cancer, vascular, pulmonary, infectious, and accidental conditions) after adjusting for demographics, systemic and ocular comorbidities, alcohol intake, smoking status, BMI, and physical activity [10]. In the present study, cataract surgery was associated with an increased cause-specific mortality (infectious and trauma or accidental). Given that cataract-related vision impairment is associated with an increased incidence of fall and fracture, and cataract surgery showed a reduced risk of fracture and accidents, protective association can be attributed to reductions in fracture and accidents after cataract surgery [28,29,30,31,32]. Furthermore, a recent meta-analysis reported that the first cataract surgery reduced the frequency of falls in older people [33]. However, several studies that evaluated interventions for preventing falls in elderly patients reported that the relationship of vision improvement after cataract surgery with reduced accidents and falls is uncertain [34,35,36]. Moreover, our observed increases in the HR might be the result of individually postponing surgery until a time point where the HR increases, in addition to the general effects of covariates [10].

Our study had some limitations. First, this study was mainly limited by its observational nature. Second, as this study was based on data from a medical insurance claims database, the diagnostic accuracy of cataract cannot be guaranteed. The identification of patients with cataract surgery, systemic comorbidities, and ocular comorbidities, using healthcare claims and Korean Standard Classification of Diseases (KCD) and Korean Electronic Data Interchange (KEDI) codes, might be inaccurate when compared with information obtained from medical charts. Moreover, the NHIS-Senior database cannot provide the information on cataract grading, objective visual acuity, axial length, presence of pseudoexfoliation syndrome, and postoperative inflammation grade. In addition, there was a lack of availability of certain covariates including metabolic profiles, BMI, alcohol intake, smoking status, and physical activity, proposing the need for further studies including various covariates. Finally, we focused only on residents of South Korea. Therefore, the observed findings cannot be generalized to other ethnic groups.

Irrespective of these limitations, this is the first report revealing the significant relationship between cataract surgery and all-cause and cause-specific mortality for the elderly population in South Korea using a nationwide, general population-based database. Another point is that this study used a large sample size of the NHIS-Senior database, and selection bias is relatively low, as the entire Korean population was enrolled in the same insurance system. In addition, we followed up the subjects until 3 years after the diagnosis of cataract without missing data because of the thorough nature of the NHIS.

## 5. Conclusions

In conclusion, we demonstrated that cataract surgery decreased all-cause and cause-specific mortality (vascular and neurologic causes) in the Korean elderly patients with cataract, especially in patients of 85 years of age and older, women, lower income, having a CCI score of 5 or more, and having no glaucoma. Even though the cataract surgery group showed lower mortality rates, it does not definitely prove a causal relationship between cataract surgery and decreased mortality, and the mechanisms underlying the relationship between cataract surgery and decreased mortality are unclear. Therefore, further longitudinal cohort studies evaluating the relationships and underlying mechanisms of the cataract surgery, systemic disease, and disease-specific mortality are needed for improving the selection of patient and the timing of cataract surgery.

## Figures and Tables

**Table 1:** Baseline characteristics of subjects according to the cataract surgery.

Variable	Total (*n* = 241,062)	Cataract Surgery Group (*n* = 127,941)	Cataract Diagnosis Group (*n* = 113,121)	ASD ^a^
Age (years)				0.1107
<70	82,011 (34.0)	44,118 (34.5)	37,893 (33.5)	
70–74	78,585 (32.6)	42,067 (32.9)	36,518 (32.3)	
75–79	47,942 (19.9)	26,228 (20.5)	21,714 (19.2)	
80–84	22,410 (9.3)	11,417 (8.9)	10,993 (9.7)	
≥85	10,114 (4.2)	4111 (3.2)	6003 (5.3)	
Mean ± SD	72.6 ± 6.1	72.4 ± 5.8	72.8 ± 6.3	0.0741
Gender				0.1175
Male	89,305 (37.0)	43,994 (34.4)	45,311 (40.1)	
Female	151,757 (63.0)	83,947 (65.6)	67,810 (59.9)	
Residence				0.0690
Metropolitan	100,398 (41.6)	51,243 (40.1)	49,155 (43.5)	
Provincial	140,664 (58.4)	76,698 (59.9)	63,966 (56.5)	
Income				0.0265
Below 20 percentiles	56,908 (23.6)	30,879 (24.1)	26,029 (23.0)	
Above 20 percentiles	184,154 (76.4)	97,062 (75.9)	87,092 (77.0)	
CCI				0.0510
0	36,616 (15.2)	18,923 (14.8)	17,693 (15.6)	
1	48,097 (20.0)	25,534 (20.0)	22,563 (19.9)	
2	45,589 (18.9)	24,754 (19.3)	20,835 (18.4)	
3	35,883 (14.9)	19,559 (15.3)	16,324 (14.4)	
4	26,200 (10.9)	14,180 (11.1)	12,020 (10.6)	
≥5	48,677 (20.2)	24,991 (19.5)	23,686 (20.9)	
Ocular comorbidity				0.2482
No	125,666 (52.1)	59,311 (46.4)	66,355 (58.7)	
Yes	115,396 (47.9)	68,630 (53.6)	46,766 (41.3)	
Severe cataract				0.2381
No	183,959 (76.3)	91,627 (71.6)	92,332 (81.6)	
Yes	57,103 (23.7)	36,314 (28.4)	20,789 (18.4)	
Glaucoma				0.1895
No	165,321 (68.6)	82,498 (64.5)	82,823 (73.2)	
Yes	75,741 (31.4)	45,443 (35.5)	30,298 (26.8)	
Age-related macular degeneration				0.0244
No	238,302 (98.9)	126,321 (98.7)	111,981 (99.0)	
Yes	2760 (1.1)	1620 (1.3)	1140 (1.0)	
DM with ophthalmic manifestations				0.0407
No	236,417 (98.1)	125,141 (97.8)	111,276 (98.4)	
Yes	4645 (1.9)	2800 (2.2)	1845 (1.6)	

CCI, Charlson Comorbidity Index; DM, diabetes mellitus; ASD, absolute standardized difference. Data are expressed as the mean ± SD, or *n* (%). ^a^ ASD of > 0.1 is considered meaningful imbalances.

**Table 2:** Mortality rates in the Korean elderly patients with cataract by surgery status.

Cause of Mortality	Mortality Rate, No. of Deaths/Total Person-Years (Incidence per 100 Person-Years; 95% CI)	
	Cataract Surgery Group	Cataract Diagnosis Group
All-cause	26,324/72,6929 (3.62; 3.58–3.67)	33,931/1,063,145 (3.19; 3.16–3.23)
Cancer	7017/726,929 (0.97; 0.94–0.99)	9415/1,063,145 (0.89; 0.87–0.90)
Vascular	6565/726,929 (0.90; 0.88–0.93)	8487/1,063,145 (0.80; 0.78–0.82)
Pulmonary	2934/726,929 (0.40; 0.39–0.42)	3408/1,063,145 (0.32; 0.31–0.33)
Neurologic	732/726,929 (0.10; 0.09–0.11)	1152/1,063,145 (0.11; 0.10–0.12)
Infectious	783/726,929 (0.11; 0.10–0.12)	815/1,063,145 (0.08; 0.07–0.08)
Accident or trauma	1768/726,929 (0.24; 0.23–0.26)	2117/1,063,145 (0.20; 0.19–0.21)

CI, confidence interval. Cox model with cataract surgery status as a time-varying covariate.

**Table 3:** Hazards of total and cause-specific mortality in the Korean elderly patients with cataract by surgery status.

Cause of Mortality (No. of Participants)	Unadjusted Cox Model Hazard Ratio (95% CI) ^a^	*p*-Value	Adjusted Cox Model Hazard Ratio (95% CI) ^a,b^	*p*-Value	Adjusted Cox Model Hazard Ratio (95% CI) ^a,c^	*p*-Value
All-cause	1.03 (1.01–1.05)	<0.001	0.95 (0.94–0.97)	<0.001	0.93 (0.92–0.95)	<0.001
Cancer	1.04 (1.01–1.08)	0.010	1.01 (0.98–1.04)	0.545	1.00 (0.97–1.03)	0.925
Vascular	1.02 (0.98–1.05)	0.337	0.93 (0.90–0.96)	<0.001	0.92 (0.89–0.95)	<0.001
Pulmonary	1.10 (1.04–1.15)	<0.001	1.01 (0.96–1.06)	0.726	0.98 (0.93–1.03)	0.358
Neurologic	0.71 (0.65–0.78)	<0.001	0.66 (0.60–0.72)	<0.001	0.64 (0.58–0.71)	<0.001
Infectious	1.25 (1.13–1.38)	<0.001	1.15 (1.04–1.28)	0.005	1.12 (1.01–1.24)	0.034
Accident or trauma	1.19 (1.12–1.27)	<0.001	1.14 (1.06–1.21)	<0.001	1.10 (1.03–1.17)	0.006

^a^ Cox model with cataract surgery status as a time-varying covariate. ^b^ Adjusted for age and sex. ^c^ Adjusted for age, sex, income, region, Charlson Comorbidity Index (0, 1, 2, 3, 4, ≥5), glaucoma, age-related macular degeneration, DM with ophthalmic manifestations, and cataract severity.

**Table 4:** Hazards of mortality in patients with cataract surgery versus cataract diagnosis by age, gender, residence, income, Charlson comorbidity index score, and ocular comorbidities in the Korean elderly patients with cataract.

Variable	Adjusted Hazard Ratio (95% CI)	*p*-Value	*p*-Value for Interaction
Age (years)			<0.001
<70	1.05 (1.02–1.09)	0.005	
70–74	0.98 (0.95–1.01)	0.238	x
75–79	0.92 (0.89–0.95)	<0.001	
80–84	0.88 (0.85–0.92)	<0.001	
≥85	0.75 (0.71–0.79)	<0.001	
Gender			<0.001
Male	1.00 (0.97–1.02)	0.717	
Female	0.88 (0.86-0.90)	<0.001	
Residence			0.831
Metropolitan	0.93 (0.91–0.95)	<0.001	
Provincial	0.93 (0.91–0.95)	<0.001	
Income			0.006
Below 20 percentiles	0.90 (0.87–0.93)	<0.001	
Above 20 percentiles	0.94 (0.93–0.96)	<0.001	
CCI			<0.001
0	1.00 (0.96–1.05)	0.908	
1	1.01 (0.97–1.05)	0.625	
2	0.99 (0.95–1.03)	0.741	
3	1.01 (0.97–1.06)	0.579	
4	0.94 (0.90–0.99)	0.018	
≥5	0.80 (0.78–0.83)	<0.001	
Severe cataract			0.276
No	0.93 (0.91–0.94)	<0.001	
Yes	0.95 (0.92–0.98)	0.001	
Glaucoma			<0.001
No	0.91 (0.89–0.93)	<0.001	
Yes	0.98 (0.95–1.01)	0.248	
Age-Related Macular Degeneration			0.279
No	0.93 (0.92–0.95)	<0.001	
Yes	1.05 (0.84–1.31)	0.661	
DM with Ophthalmic Manifestations			0.820
No	0.93 (0.92–0.95)	<0.001	
Yes	0.94 (0.86–1.03)	0.203	

CCI, Charlson Comorbidity Index; DM, DM, diabetes mellitus. Cataract diagnosis group was used as a reference in all models. Adjusted for age, gender, income, region, Charlson Comorbidity Index (0, 1, 2, 3, 4, ≥5), glaucoma, age-related macular degeneration, DM with ophthalmic manifestations and cataract severity.

## Data Availability

The data that support the findings of this study are available from NHIS, but restrictions apply to the availability of these data, which were used under license for the current study and thus are not publicly available. However, data are available from the authors upon reasonable request and with permission of NHIS.

## Supplementary Materials

The following are available online at https://www.mdpi.com/article/10.3390/jpm11111128/s1, Table S1: Korean Standard Classification of Diseases (KCD) codes and Korean Electronic Data Interchange (KEDI) codes included in definition of cataract and cataract surgery, Table S2: Korean Standard Classification of Diseases (KCD) codes included in mortality attributed to specific systemic conditions, Table S3: Korean Standard Classification of Diseases (KCD) codes included in the definition of comorbidities.
