# Supplementary material for: Relationship between Cataract Surgery and Mortality in Elderly Patients with Cataract: Nationwide Population-Based Cohort Study in South Korea
Source: J Pers Med. 2021 Nov 1;11(11):1128. doi: 10.3390/jpm11111128 (PMC8625327; doi:10.3390/jpm11111128)
Supplement: Supplementary file 1 [file jpm-11-01128-s001.zip › jpm-1428899-supplementary.pdf]

Table S1. Korean Standard Classification of Diseases (KCD) codes and Korean Electronic Data Interchange (KEDI) codes included in definition of cataract and cataract surgery.

Condition	Eligible KCD Diagnosis Code
Cataract	H25.0, H25.1, H25.2, H25.8, H25.9, H26.02, H26.21, H26.28, H26.3, H26.8, H26.9, H28.0, H28.1, H28.2
Severe cataract	H25.1, H25.2
**Cataract surgery**	**Eligible KEDI Code**
extracapsular or intracapsular extraction	S5111
phacoemulsification	S5119
primary intraocular lens implantation	S5117

KCD, Korean Standard Classification of Diseases; KEDI, Korean Electronic Data Interchange.

Table S2. Korean Standard Classification of Diseases (KCD) codes included in mortality attributed to specific systemic conditions.

Type of Mortality	Eligible KCD Diagnosis Code
Cancer death	C00-C14, C15-C26, C30-C39, C40-C41, C43-C44, C45-C49. C50, C51-C58, C60-C63, C64-C68, C69-C72, C73-C75, C76-C80, C81-C96, C97-C97, D00-D09, D10-D36, D37-D48
Vascular death	I00-I02, I05-I09, I10-I15, I20-I25, I26-I28, I30-I52, I60-I69, I70-I79, I80-I89, I95-I99
Pulmonary death	J00-J06, J09-J18, J20-J22, J30-J39, J40-J47, J60-J70, J80-J84, J85-J86, J90-J94, J95-J99
Neurologic death	G00-G09, G10-G14, G20-G26, G30-G32, G35-G37, G40-G47, G50-G59, G60-G64, G70-G73, G80-G83, G90-G99
Infection-related death	A00-A09, A15-A19, A20-A28, A30-A49, A50-A64, A65-A69, A70-A74, A75-A79, A80-A89, A90-A99, B00-B09, B15-B19, B20-B24, B25-B34, B35-B49, B50-B64, B65-B83, B85-B89, B90-B94, B95-B98, B99
Accident or trauma related death	V01-V99, W00-W99, X00-X84, X85-Y09, Y10-Y34, Y35-Y36, Y40-Y59, Y60-Y69, Y70-Y82, Y83-Y84, Y85-Y89, Y90-Y98

KCD, Korean Standard Classification of Diseases.

Table S3. Korean Standard Classification of Diseases (KCD) codes included in the definition of comorbidities.

Comorbidity	Eligible KCD Diagnosis Code
Lymphomas	C81-C86, C88
Multiple myeloma/leukemia	C90-C96
Malignant neoplasms	C00-97
Myocardial infarction	I21-I23
Heart failure	I50, I97.1, I09.9, I11.0, I13.0, I13.2
Cerebrovascular disease	I60-I69

Peripheral vascular disease	I73
Chronic pulmonary disease	J44, I27.9, J84
Cirrhosis	K70.3, K71.7, K74, K76.1
Hepatic failure	K70.4, K71.1, K72
AIDS	B20-B24
Hemi/paraplegia	G04.1, G11.4, G81-82, I69.006, I69.106, I69.206, I69.306, I69.406
Rheumatologic disease	M05, M06, M10, M12.0, M12.3, M30-M36
Dementia	F00-F03, F05.1, G31.82
Diabetes mellitus	E10-E14
Diabetes mellitus with complications	E10.0-E10.8, E11.0-E11.8, E12.0-E12.8, E13.0-E13.8, E14.0-E14.8
Chronic renal disease	E10.22, E12.22, E13.22, E14.22, I12, I13
Peptic ulcer disease	K27
Glaucoma	H40, H42
Age-related macular degeneration	H35.30, H35.31, H35.39
Diabetes mellitus with ophthalmic manifestations	E10.3[†], E11.3[†], E12.3[†], E13.3[†], E14.3[†]

KCD, Korean Standard Classification of Diseases; AIDS, Acquired immune deficiency syndrome.
[†]1,2,3,4,8.
